# Hand and Arm Bimanual Intensive Therapy Including Lower Extremities (HABIT-ILE@home) for Adults With Chronic Stroke: Protocol for a Randomized Controlled Trial

**DOI:** 10.2196/87035

**Published:** 2026-02-10

**Authors:** Merlin Somville, Zélie Rosselli, Edouard Ducoffre, Carlyne Arnould, Yannick Bleyenheuft, Geoffroy Saussez

**Affiliations:** 1 Institute of NeuroSciences (IoNS), UCLouvain, Avenue Emmanuel Mounier 83, Bruxelles, 1200, Belgium, 32 2 764 54 46; 2 CeREF-Santé, Haute École Louvain en Hainaut, Montignies-sur-Sambre, Belgium

**Keywords:** chronic stroke, intensive rehabilitation, motor function, motor skill learning, bimanual therapy, HABIT-ILE, home-based training, telerehabilitation, caregiver involvement, randomized controlled trial, hand and arm bimanual intensive therapy including lower extremities

## Abstract

**Background:**

Intensive therapies based on motor skill learning have been widely used in stroke rehabilitation for improving upper extremity abilities, demonstrating significant improvements in arm function and daily life activities. Based on the same therapeutic principles of motor skill learning, hand and arm bimanual intensive therapy including lower extremities (HABIT-ILE) was developed focusing on bimanual coordination and constant concomitant stimulation of trunk control and lower extremities. However, the implementation of such high-dosage interventions in stroke rehabilitation might face barriers due to limited accessibility and high resource requirements. Delivering HABIT-ILE therapy at home (HABIT-ILE@home) via telerehabilitation may reduce logistical barriers while maintaining efficacy. In addition, the added value of a 9-week specific follow-up program will be tested after high dosage interventions.

**Objective:**

The first randomized controlled trial (RCT1) aims to evaluate the noninferiority of a high-dosage HABIT-ILE@home program compared to its on-site counterpart. The second randomized controlled trial (RCT2) aims to test the superiority of a 9-week specific HABIT-ILE@home follow-up vs a nonspecific home program.

**Methods:**

A total of 48 adults with chronic stroke will be randomized to either HABIT-ILE@home or HABIT-ILE on-site group (RCT1, 65 hours over 2 weeks). HABIT-ILE@home will follow the same principles as HABIT-ILE on-site but will be delivered by caregivers with remote supervision by trained therapists and the use of a dedicated telerehabilitation device to facilitate intervention delivery and remote monitoring (ie, REAtouch Lite; Axinesis). All participants will then participate in a HABIT-ILE@home follow-up program or nonspecific follow-up (RCT2, 45 hours over 9 weeks). Primary outcomes will be the change in Fugl-Meyer Assessment (FMA), while secondary outcomes include feasibility and adherence questionnaires, upper and lower extremity motor function assessments, daily activities, and quality of life questionnaires. Assessments will be performed before (T0) and after (T1) the 2 weeks of high dosage intervention, followed by an assessment after the 9-week follow-up (T2).

**Results:**

Recruitment for the trial started in March 2023 and ended in March 2025, and data collection has been completed for this study. Data analysis is planned to start early 2026; we expect to submit the results for publication in spring 2026.

**Conclusion:**

This study will provide evidence on the feasibility and efficacy of delivering HABIT-ILE through a home-based telerehabilitation model for adults with chronic stroke. Demonstrating noninferiority of HABIT-ILE@home compared to on-site therapy would support wider accessibility to intensive rehabilitation while reducing logistical and human resource constraints. Additionally, showing the added benefit of a structured follow-up could emphasize the importance of continuity of care to sustain and enhance motor recovery after intensive interventions.

## Introduction

### Background

The global lifetime risk of stroke in the adult population is 24.9% [1]. Stroke is the second leading cause of death and the primary etiology of motor disability in adults worldwide [2]. After a stroke, patients experience variable acute symptoms depending on the type, location, and size of the brain lesions: hemiparesis, which often causes partial or complete inability to use one upper limb, spasticity, loss of independent walking, chronic pain, alterations of sensations, and visuospatial neglect [3,4]. It is important to note that some of these symptoms may become chronic. The long-term consequences of the pathology, particularly the motor impairments, can lead to difficulties in daily activities and a lower quality of life [5,6].

Rehabilitation plays an important role in reducing the impact of symptoms and in improving the quality of life of stroke patients. A wide variety of rehabilitation interventions are available, including strength or manual dexterity training, neuromuscular stimulation, balance exercises, walking and task training, and stretching of the affected side [7]. The most effective interventions identified in the literature are those based on the principles of motor skill learning, such as constraint-induced movement therapy (CIMT), hand-arm bimanual intensive training, bilateral arm training, and high dose repetitive task practice [8-11]. These therapies have demonstrated significant improvement in upper extremity functions and activities of daily living [12-15]. More recently, hand and arm bimanual intensive therapy including lower extremities (HABIT-ILE) has been developed with a focus on bimanual coordination training with stimulation of postural control and lower extremities [16]. In adults with stroke, the implementation of a HABIT-ILE training is under investigation through a randomized controlled trial with expectations of similar improvements [17]. Although no results on the efficacy of HABIT-ILE in stroke patients are currently available, improvements similar to those observed in upper extremities with other intensive therapies based on motor skill learning are expected.

HABIT-ILE is usually organized through a 2-week on-site camp. Although this high therapeutic dosage is one of the ingredients necessary for its efficacy, it can also be considered an obstacle to the implementation of HABIT-ILE in clinical practice. As the number of locations offering HABIT-ILE therapy is currently limited, patients and their families often have to travel long distances to reach the therapy site and find accommodation for the duration of the treatment, and those unable to meet these burdens will not have access to therapy. The organization of HABIT-ILE sessions on-site is also challenging, as it requires specialized facilities, extensive equipment, and a large number of trained therapists (ie, one per participant), in a context where there is a lack of therapists qualified to administer this type of therapy. Additionally, public health crises such as the COVID-19 pandemic have demonstrated how vulnerable such therapy models can be, as they can be significantly disrupted by global health emergencies. These constraints are not specific to HABIT-ILE but are shared by other intensive motor skill learning-based interventions and contribute to limiting their dissemination in routine practice [18,19]. To overcome these potential barriers and improve the accessibility of HABIT-ILE, we propose a telerehabilitation modality.

Besides intensive interventions, access to outpatient therapies after stroke is sometimes limited, especially in rural areas [20]. Although some motor skills acquired during intensive motor skill learning therapies are maintained in the long term [21], some functional goals or newly acquired motor abilities might be difficult to transfer to daily life directly after a camp. The long-term and additional effects of such intensive interventions could be enhanced with a specific follow-up intervention focusing on the potentiation of the newly acquired abilities and their transfer in daily life. Based on this assumption, we aim to investigate the efficacy of a follow-up program of HABIT-ILE therapy at home (HABIT-ILE@home) in adults with stroke after the completion of intensive HABIT-ILE intervention (either on-site or at home).

### Aims and Hypotheses

This protocol comprises 2 randomized controlled trials (RCTs), hereafter referred to as RCT1 and RCT2, conducted in the same cohort of 48 adults with chronic stroke. The first trial (RCT1) aims to test whether a 2-week high-dosage HABIT-ILE telerehabilitation program (HABIT-ILE@home) is noninferior to a 2-week high-dosage on-site HABIT-ILE camp in improving motor function. We hypothesize that both interventions will lead to significant improvements in motor function, activities, and participation, and that the HABIT-ILE@home group will be noninferior to the on-site group when comparing changes from baseline to postintervention (T1-T0). We further hypothesize that HABIT-ILE@home will demonstrate higher self-perceived burden for caregivers but will show acceptable feasibility, adherence, and perceived efficacy.

The second trial (RCT2) aims to evaluate whether a structured 9-week specific HABIT-ILE@home follow-up program is more effective than a dose-matched nonspecific home-based follow-up in maintaining and further enhancing the improvements obtained after the intensive phase. We hypothesize that, over the follow-up period (T2-T1), participants allocated to the specific HABIT-ILE@home follow-up will show superior retention and/or additional improvements in motor function, activity performance, and social participation compared with those receiving the nonspecific follow-up. We further hypothesize that HABIT-ILE@home follow-up will demonstrate higher self-perceived burden for caregivers but will show acceptable feasibility, adherence, and perceived efficacy. All outcomes will be assessed at baseline (T0), immediately after the intensive 2-week intervention (T1), and after the 9-week follow-up period (T2).

## Methods

### Ethical Considerations

The study was approved by the Hospital-Faculty Ethics Committee of Saint-Luc – UCLouvain, Belgium (clinical trial number: B4032022000142) and was registered on ClinicalTrials.gov (NCT05727111) on February 2, 2023. Participants were informed by means of a comprehensive information document and signed an informed consent form. All data collected for this study will be treated anonymously. Compensation for research-related harm, if applicable, will be covered according to the institutional insurance arrangements in place for this study. Written consent for the use of data, including images and videos, was obtained from the participants, or from their parents or legal guardians in the case of minors. The data will be anonymized using allocation codes for analysis after both RCTs.

### Study Design

This study consists of 2 sequential, 2-arm, parallel-group pragmatic, single-blind RCTs conducted within the same cohort of adults with chronic stroke. RCT1 is a noninferiority trial comparing a 2-week high-dosage HABIT-ILE@home program with a dose-matched on-site HABIT-ILE camp (allocation ratio 1:1). RCT2 is a superiority trial comparing a 9-week specific HABIT-ILE@home follow-up to a dose-matched non-specific home-based follow-up. Randomization will be performed using pairwise randomization, and outcome assessors are blinded to group allocation. All evaluations will occur at baseline (T0), postintensive intervention (T1), and after the follow-up period (T2).

### Patient and Public Involvement

Before this RCT, a pilot HABIT-ILE@home study was conducted in which participants and caregivers completed questionnaires on feasibility, acceptability, and burden; their feedback was used to modify the current study protocol and enhance several components of the HABIT-ILE@home intervention. In the present trial, participants’ perception is gathered through completing satisfaction and burden questionnaires at the end of each intervention phase.

### Participants and Setting

**Figure 1.**
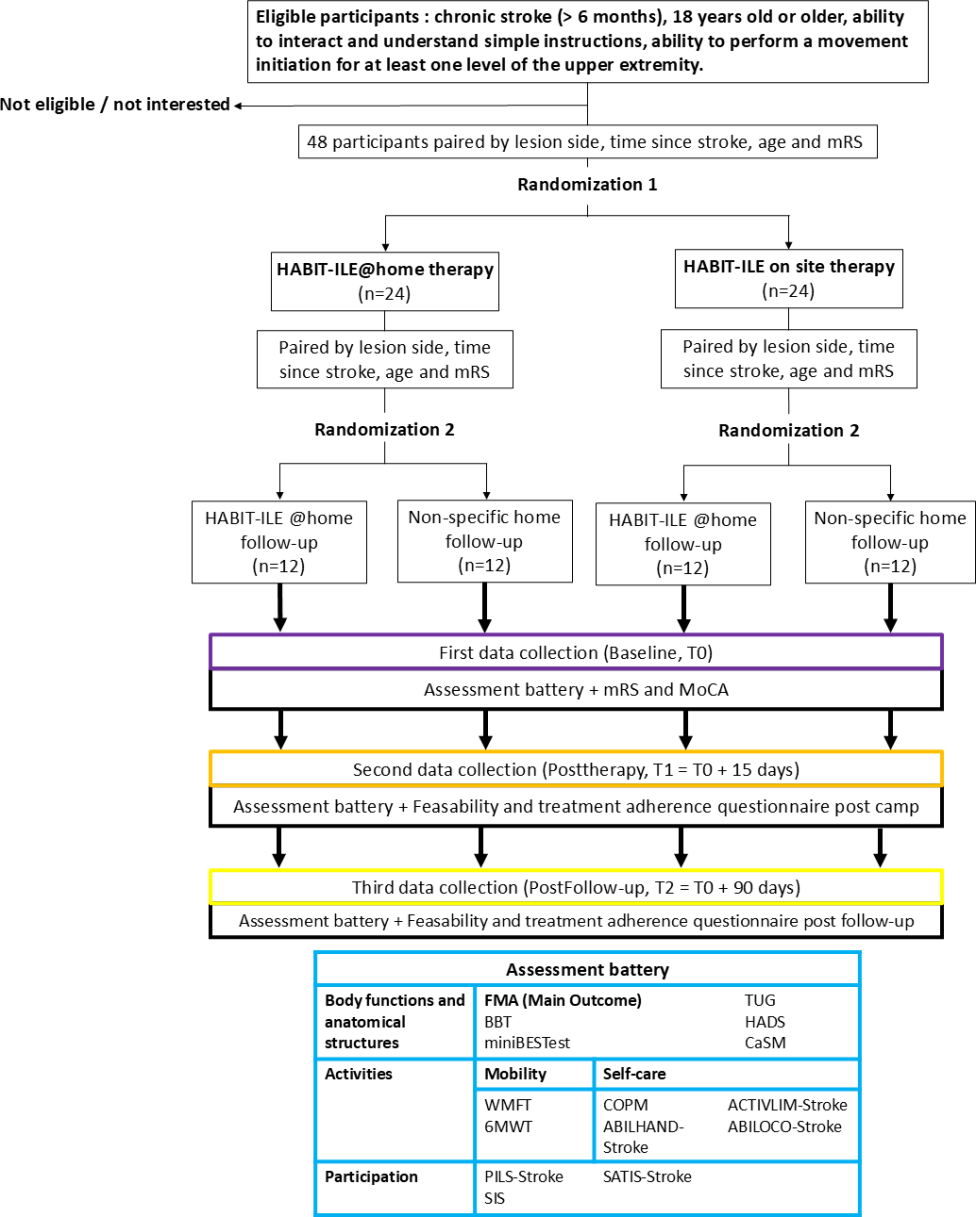
CONSORT (Consolidated Standards for Reporting Trials) flowchart. ACTIVLIM: Activity Limitations Questionnaire; 6MWT: 6-minute walk test; BBT: box and block test; CaSM: Confidence After Stroke Measure; COPM: Canadian Occupational Performance Measure; FMA: Fugl-Meyer Assessment; HADS: Hospital Anxiety and Depression Scale; Mini-BESTest: Mini Balance Evaluation Systems Test; MoCA: Montreal Cognitive Assessment; mRS: modified Rankin scale; PILS-Stroke: Participation In Life Situations - Stroke; SIS: Stroke Impact Scale; TUG: timed up and go test; WMFT: Wolf Motor Function Test.

A total of 48 participants with chronic stroke will be recruited from Belgium and surrounding countries through mailing, spontaneous applications, social networks, physicians, physiotherapists, occupational therapists, and hospitals. Participants will be considered eligible if they meet the following criteria: time since stroke of more than 6 months, ages 18 years and older at inclusion, ability to interact, and understand simple instructions for completing assessments and therapy, ability to voluntarily initiate minimal movement of the affected upper extremity (eg, observable slight minimal movement in either shoulder, elbow, wrist, or fingers). A caregiver must also be available for 6.5 hours per day over 2 weeks for the needs of the high-dosage HABIT-ILE@home intervention (RCT1) and for the duration of follow-up programs (RCT2). Participants will be ineligible if one of the following criteria is present: botulinum toxin injections, orthopedic surgery, or another intensive therapy during the 3 months before the first assessment (T0) or during the study period. Additionally, participants will not be included if they are pregnant or present uncontrolled seizures or other uncontrolled health issues such as heart or renal failure. Cognitive function will be assessed at baseline using the Montreal Cognitive Assessment. This measure will only serve as a descriptor, not for eligibility or outcomes. All assessments will be conducted on the UCLouvain premises in Brussels or Louvain-la-Neuve. This study will be reported following CONSORT guidelines (Figure 1).

### Randomization Process

For RCT1, randomization will be performed using pairwise randomization. Participants will first be paired according to lesion side, time since stroke, age, and modified Rankin Scale (mRS) level. Participants in each pair will then be randomized into one of the two groups using an online external blind electronic randomization system [22], which will provide the assignment only after pairing is completed. This procedure will ensure allocation concealment, as investigators will not be able to predict or influence the upcoming allocation. For RCT2, the same procedure will be repeated within each RCT1 allocation arm, such that participants previously assigned to HABIT-ILE@home or HABIT-ILE on-site will be again matched and randomized in the HABIT-ILE@home follow-up subgroup or the nonspecific follow-up subgroup.

### Sample Size

For RCT1, assessing the noninferiority of HABIT-ILE@home compared to HABIT-ILE on-site, the sample size was calculated using PASS (version 14.0.15; NCSS) for a noninferiority 2-tailed *t* test of the difference between two means. The primary outcome is the change in the upper extremity subscale of the Fugl-Meyer Assessment (FMA-UE) score from baseline to postintervention (T1-T0), with higher scores indicating better motor abilities. We defined the noninferiority margin (NIM) using a method that ensures both clinical and statistical relevance, rather than only relying on the minimal clinically important difference. We chose the NIM as 50% of the difference between the changes observed with the gold-standard treatment and an interventional control group. This method was chosen to provide a more stringent criterion for noninferiority, ensuring that any treatment deemed non-inferior demonstrates a real benefit for stroke patients. As no previous studies have investigated such changes after a HABIT-ILE intervention, the sample size calculation was based on the mean change observed after a CIMT intervention (+5.6 points at FMA-UE; SD 3.1) [13]. The non-inferiority hypothesis was defined as follows: the HABIT-ILE@home intervention will be considered noninferior if the improvement produced is no more than 2.8 points lower than that obtained with the on-site HABIT-ILE intervention on the FMA-UE. An α level of 0.05 and a 1-β level of 0.8 were used for calculation. Under these assumptions, sample size calculation defined a number of 17 participants per group, resulting in a total of 34 participants.

For RCT2, which assesses the superiority of the specific HABIT-ILE@home follow-up compared with the nonspecific follow-up, the sample size calculation was also based on the mean changes in the FMA-UE observed in the study of Bonifer et al [13] by adjusting the expected improvements to account for the lower number of therapy hours in RCT2. Scaled proportionally, a 45-hour program corresponds to an expected improvement of 2.8 points. Because both follow-up arms constitute active interventions, the superiority margin was set at 1.4 points (50% of the expected 45-hour gain), reflecting a meaningful difference between two active interventions. Consequently, 17 participants are needed per group, resulting in a total of 34 participants, with a α level of .05 and a 1-β level of .8. To ensure that the required number of participants complete both phases, we plan to recruit 48 individuals, corresponding to an anticipated attrition rate of about 30%. This conservative estimate was based on a previous study with a similar population and reflects the two-phase design and the substantial differences between intervention modalities, with participants enrolled before being informed of their allocation [17].

### Blinding Procedure

The main outcome (FMA) will be video-recorded and scored by experienced external raters, who are blind to group allocation and timing of assessment. Regarding secondary outcomes, the videos of the Wolf Motor Function Test (WMFT) and Mini Balance Evaluation Systems Test (Mini-BESTest) will also be scored blindly by external raters. As mentioned, the data will be anonymized using allocation codes for analysis after the end of both RCTs.

### Study Interventions

#### RCT 1: HABIT-ILE@home vs HABIT-ILE on-Site Camp (2 Weeks)

##### Overview

During the first RCT, all participants will complete 2 weeks of HABIT-ILE therapy either at home or in an on-site camp. All the participants will benefit from 65 hours of intervention over 2 weeks (6.5 hours per day, 5 days per week).

HABIT-ILE is an intensive therapy that involves both hands and systematically engages the trunk and/or lower extremities in various functional activities [16,17]. It is based on the key therapeutic principles of motor skill learning [23,24]. HABIT-ILE is a goal-directed intervention, focusing on self-determined goals (maximum 5 goals/participant). It involves massed practice, which includes a high dosage of therapy, repetition of movements (trial-error training), and a high motor engagement time (ie, the percentage of time during which participants practice active movements). Therefore, the stimulation of voluntary motor control is achieved through hands-off practice. Throughout the intervention, the difficulty of the proposed structured activities is gradually increased based on motor, environmental, or task components. Participants’ motivation is supported, notably through playful activities based on the patients’ interests. For more details about the HABIT-ILE therapy, see Bleyenheuft and Gordon [16] and Ebner-Karestinos et al [17].

##### HABIT-ILE On-Site Camp (Control Group)

HABIT-ILE on-site intervention will be held in a rehabilitation center in Belgium. All participants will be in the same room. Each participant will benefit from an individualized intervention with one or two dedicated interventionists (occupational therapists, physiotherapists, or students of occupational therapy or physiotherapy) during the whole process. At the end of each day, a group activity will be performed with all the participants.

The therapeutic plan of each participant will be established, supervised, and adapted day after day by a team of supervisors (occupational therapist and/or physiotherapist specifically trained for the supervision of a HABIT-ILE intervention). The supervisors will coach the interventionists throughout the day to ensure the application of HABIT-ILE and motor skill learning principles. In addition, at the end of each treatment day, the supervision team will discuss the session and coach the interventionists to adapt the therapeutic plan and define the type of activities for the next day, in order to ensure goal-directed motor learning throughout the intervention.

##### HABIT-ILE@home (Treatment Group)

During the HABIT-ILE@home intervention, all participants will perform the intervention at home, accompanied by a non-therapist caregiver (patient’s relative, partner, friends, etc). The caregiver will be coached by a supervision team through remote supervision to monitor the delivery of the intervention. The supervision team will include 1 to 2 supervisors and trained therapists, with each trained therapist coaching up to 4 patients during the intervention.

The caregiver will be involved throughout the entire therapeutic process to observe movement parameters, identify compensatory movements, and adapt the therapeutic environment in line with the “hands-off” and “increasing difficulty” principles (eg, by modifying the type of manipulated objects, adjusting the spatiotemporal requirements of the task, the role of the hands, and the standing or sitting environment). The caregiver will also provide positive feedback, motivate the patient, and ensure safety.

To fulfill their role successfully, caregivers will receive support from the supervision team in various ways. Before the therapy, caregivers will receive an adapted HABIT-ILE@home training session (one hour) as well as a “caregiver manual” to explain: (1) how to apply HABIT-ILE intervention and motor skill learning therapeutic principles, (2) their role as a caregiver, and (3) the rehabilitation equipment loaned to the participants. Two extra training hours will be provided the week before the therapy to familiarize participants with the telerehabilitation system used throughout the intervention. Moreover, the supervision team will generate and provide a customized treatment schedule to caregivers each evening for the next therapeutic day. Each program will include a description of the trained movements and skills as well as information about the proposed task-oriented activities or games (type of objects, type of grasping, orientation of objects, types of games, and duration).

**Figure 2.**
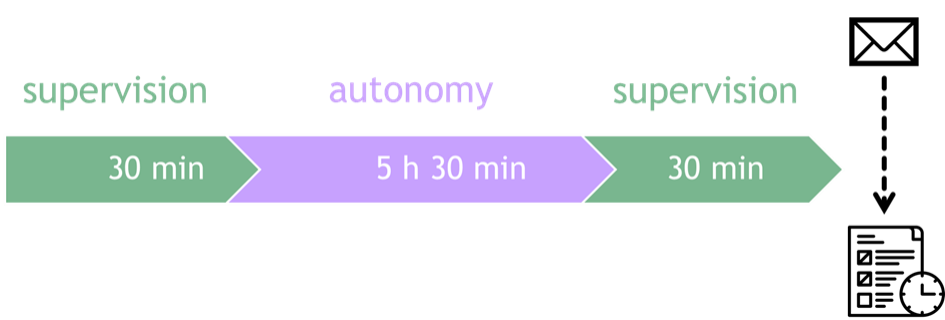
Course of a HABIT-ILE@home therapy day: A day of HABIT-ILE@home will begin with 30 minutes of remote supervision via video conferencing software, then the participants and their caregivers will be left for 5 hours and 30 minutes in autonomy. Finally, the day will end with another 30 minutes of remote supervision. At the end of the day, the supervisor will send the treatment schedule for the next day.

Remote supervision will be scheduled for one hour each day within the 6.5 hours of therapy (Figure 2). The supervision sessions will be divided into two 30-minute sessions, one at the beginning of each therapy day, and one at the end. The first 30-minute session will be used to explain the daily therapeutic plan, demonstrate how to set up and carry out goal-oriented activities (instructions regarding the trained movements, the way to present manipulated objects, ensure safety, the type of instructions, and feedback needed) and adapt the therapeutic environment. Note that this initial session will last 1 hour on the first training day. The second 30-minute end session will be used to address participants’ questions, discuss any difficulties encountered, assess the participants’ progress, test new activities to adjust the difficulty level, and plan the therapeutic program for the following day.

The implementation of HABIT-ILE therapy at home will be facilitated using a telerehabilitation device, the REAtouch Lite (Axinesis). REAtouch Lite is a smaller and lighter version of the REAtouch device, which was previously used in HABIT-ILE on-site camps with a pediatric population [25]. The REAtouch device is an interactive interface that interacts with customizable tangible objects through a range of virtual games and activities. It offers an environment designed to assist clinicians or caregivers in motivating the patient throughout the intervention and facilitate the decision-making process for implementing motor skill learning principles [25]. Note that, depending on the patients’ therapeutic needs, goals, and the course of the intervention, activities will be performed with or without direct use of the REAtouch Lite. In addition to being a smaller and more transportable version of the initial device, the REAtouch Lite is equipped with a telecommunication module designed for remote communication and rehabilitation, including a smartphone for directly interacting with the patients and caregivers during supervision sessions. The use of a smartphone will enable supervision sessions both inside and outside the patient’s home, and the caregiver will be able to take photos and videos and send them easily to the supervision team. The smartphone will be supplied with three phone holders to easily adjust camera angles during sessions; position 1: in front of the REAtouch Lite, providing a precise view of the patient. Position 2: positioned to the side of the patient, focusing on the lower limbs. Position 3: positioned for a wider perspective, allowing observation of movements within a 3 meter range.

**Figure 3.**
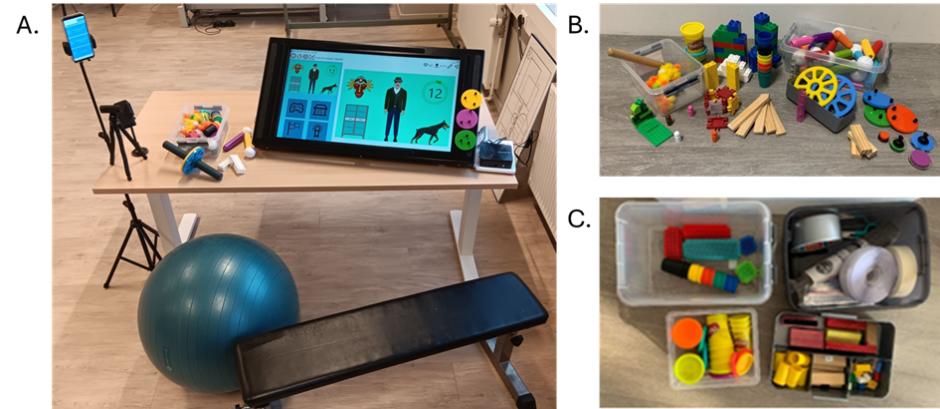
Therapeutic environment delivered for HABIT-ILE@home, including: (A) an illustration of delivered therapeutic environment: phone holders, the Reatouch Lite, gym ball and/or adjustable bench, an adjustable table; (**B**) objects of various shapes and sizes to elicit different types of grasping; (**C**) adaptation material, such as velcro straps, tape, nonslip mat, etc, along with objects delivered.

Moreover, in the context of the HABIT-ILE@home program, the REAtouch Lite device will be supplied with a table with adjustable height to ensure adaptability based on postural control and lower extremities involvement. The therapeutic environment will be enhanced by the addition of an adjustable bench, an inflatable ball, and/or a standing balance board, depending on the participants’ motor abilities and functional goals. Furthermore, a box containing therapeutically useful objects and materials for adaptations (such as Velcro straps, tape, etc) will be delivered alongside the REAtouch Lite device (Figure 3).

### RCT 2: HABIT-ILE@home vs Nonspecific Follow-Up Interventions (9 Weeks)

#### Overview

During RCT2, all participants will take part in a low- to moderate-dosage home follow-up intervention, either with a specific HABIT-ILE@home program or with a nonspecific follow-up program managed by patients’ usual therapists. In both groups, the follow-up program will last 9 weeks, with a recommendation of 5 hours of therapy per week (in addition to usual care) performed at home (45 hours in total).

#### Specific HABIT-ILE@home Follow-Up (Treatment Group)

For the specific HABIT-ILE@home follow-up, the treatment modalities will be the same as for the 2-week HABIT-ILE@home intervention described previously, except for the therapeutic and supervision dosage. A 1-hour session will be planned each week, as part of the 5 recommended hours. Like the previously mentioned daily program, a detailed therapeutic schedule will be sent and adapted every 1 or 2 weeks, depending on the participants’ needs.

#### Nonspecific Home Follow-Up (Control Group)

For the nonspecific home follow-up, participants’ usual therapist will be contacted and asked to develop a 5-hour weekly program based on their usual practice and treatment plan for 9 weeks. If contact could not be established with the participants’ usual therapists, documentation with instructions for self-rehabilitation exercises will be sent to the patients and their caregivers, including stretching of the affected side, muscle strengthening, and task-oriented exercises as described in an online booklet [26].

### Criteria for Discontinuing or Modifying Allocated Interventions

The intervention may be discontinued at the participant’s request or if safety/tolerability concerns arise. All reasons will be documented, and follow-up assessments will be completed whenever possible.

### Data Collection and Data Management

After each assessment session, data will be entered into the Research Electronic Data Capture (REDCap) system hosted at UCLouvain. A detailed codebook will be created to document each variable, including its name, possible values, data labels, and units.

### Data Monitoring Committee

At UCLouvain, there is no data monitoring committee for nondrug interventional studies. However, the Hospital-Faculty Ethics Committee of Saint-Luc, composed of a majority of doctors (from UCLouvain or the Cliniques Universitaires de Saint-Luc) but also including a general practitioner, nurses, pharmacists, lawyers, ethicists, a methodologist, a psychologist, scientific collaborators, patients’ representatives, and healthy volunteers’ representatives, ensures that participants in the clinical trials are not exposed to undue risks. The Ethics Committee reviews the study protocol and all relevant information about the experimental treatment to ensure research safety. Additionally, it verifies that the information provided to participants is clear and accessible, enabling fully informed consent. Importantly, the Committee operates independently from the study sponsor, with no members affiliated with the research project.

### Outcomes

#### Primary Outcomes

The primary outcome measure is change in the FMA. The FMA is a standardized assessment tool initially designed to measure motor function, sensation (light touch and position sense), balance, joint range of motion, and joint pain in the upper and lower extremities of poststroke patients [27]. More specifically, the motor function subsection assesses reflex activity, volitional movement, and coordination/speed. Only the total motor score (upper and lower extremities) will be used in this study. The score varies from 0 to 100 points, with 66 points for upper extremities and 34 for lower extremities. Higher values represent better motor function. The FMA has excellent reliability [28], and its validity [29] and responsiveness [30] have been demonstrated. The training procedure proposed to enhance standardization of the assessment [31] will be completed by both examiners and blind assessors. For RCT1, the primary outcome for comparison will be the change from T0 to T1, and for RCT2, the primary outcome for comparison will be the change from T1 to T2.

#### Secondary Outcomes

The same secondary outcome battery will be administered at T0, T1, and T2 for both RCT1 and RCT2. For RCT1, secondary outcomes for comparison will be the change from T0 to T1, and for RCT2 the secondary outcomes will be the change from T1 to T2.

##### Body Structures and Functions

Anxiety and depression will be measured by the Hospital Anxiety and Depression Scale (HADS) [32]. The HADS is a 14-item self-perceived questionnaire (7 items for the anxiety subscale and 7 items for the depression subscale) asking participants to estimate their mood in the past week [33]. Each item is rated on a 4-level scale, leading to a total score ranging from 0 to 21 points for each subscale used. Higher scores indicate higher anxiety and depression. HADS has shown an excellent internal consistency in the stroke population [34].

Gross unimanual dexterity will be assessed by the Box and Block Test, which measures the number of blocks transported from one compartment of a box to another in a minute [35]. This tool shows excellent test-retest reliability and is sensitive to detect changes in stroke patients [36,37].

##### Activities

Upper extremity motor capacity will be measured by the 17-item version of the WMFT [38]. A total of 15 items must be performed as quickly as possible, with a 120-second time limit. A total of 6 items assess joint-segment movements while 9 items evaluate integrative functional movements. Each of these 15 items is rated on a 6-level scale, leading to a total score ranging from 0 to 75 points. Higher scores represent higher upper extremity motor capacity. The remaining 2 items involve measures of strength. The WMFT is considered a reliable and valid measurement tool [38].

Manual activity performance, defined as the ability to manage daily activities requiring the use of the upper extremities (whatever the strategies involved), will be assessed using the self-administered Abilities of the Hand (ABILHAND-Stroke) questionnaire. Patients will be asked to report their ease or difficulty in performing 23 bimanual activities. As a Rasch-built questionnaire, the measures which are linear correspond to the percentage of the full range scale expressed in logits, from 0% (low manual performance) to 100% (high manual performance). This questionnaire shows a high reliability and a good validity [39].

Balance will be measured by the Mini-BESTest, which is a shortened version of the Balance Evaluation Systems Test. A total of 4 main domains of balance (anticipatory postural adjustments, reactive postural control, sensory orientation, and dynamic gait) are evaluated through 14 items. Each item is rated on a 3-level scale, leading to a total score ranging from 0 to 28 points. Higher values indicate better balance. This test is considered reliable and valid [40].

Lower extremity capacity, including mobility, balance, and walking skills, will be assessed by the timed up and go test. From a seated position, the patient will be asked to stand up, walk 3 meters, then turn around, walk 3 meters, and sit down. The score (in seconds) is the time taken to complete the functional task. The timed up and go test is a reliable and valid test for stroke patients [41].

Walking endurance will be evaluated by the 6-minute walk test [42]. The patients will have to cover the greatest possible distance in 6 minutes in a 30 m hallway, without taking a break. The 6-minute walk test is a good measure of endurance, giving an idea of daily life performance. This test has excellent validity and reliability [41].

Locomotor performance, defined as the patients’ ability to move about effectively in their environment, will be measured by the ABILOCO-Stroke questionnaire. Patients will be asked whether they are able to perform 13 locomotor activities on a 2-level scale (impossible or possible). ABILOCO-Stroke shows good psychometric properties [43]. As a Rasch-built questionnaire, the measures which are linear correspond to the percentage of the full range scale expressed in logits, from 0% (low locomotor performance) to 100% (high locomotor performance).

Global activity performance, defined as the ability to manage daily activities requiring the use of the upper and/or lower extremities (whatever the strategies involved), will be assessed by the Activity Limitations Questionnaire (ACTIVLIM-Stroke) questionnaire. Patients will be asked to report their ease or difficulty in performing 20 daily activities. As a Rasch-built questionnaire, the measures which are linear correspond to the percentage of the full range scale expressed in logits, from 0% (low global performance) to 100% (high global performance). The questionnaire shows a high validity [44].

The self-perceived patient’s performance in functional goals and their satisfaction with achieved performance will be evaluated through the Canadian Occupational Performance Measure. Patients will be asked to estimate their performance and their satisfaction on a 10-level scale, from 1 (low performance/satisfaction) to 10 (high performance/satisfaction). The Canadian Occupational Performance Measure is a reliable, valid, and responsive outcome measure [45].

Global disability level will be classified using the mRS. This scale classifies stroke patients into 7 levels, from 0 (no symptoms) to 5 (severe disability: confined to bed, incontinent, and requiring constant nursing care and attention) and 6 (death) [46]. The scale has shown excellent test-retest reliability [47].

##### Participation

Satisfaction in activities and social participation will be assessed by the SATIS-Stroke questionnaire. Patients will be asked to rate their satisfaction in the achievement of 36 activities or social life situations on a 4-level scale. As a Rasch-built questionnaire, the measures which are linear correspond to the percentage of the full range scale expressed in logits, from 0% (low satisfaction) to 100% (high satisfaction). Excellent test-retest reliability has been shown [48].

Participation, defined as the patient’s involvement in life situations (chosen because they make sense to them) in which they interact with others or which are linked to social roles, will be measured by the Participation In Life Situations – Stroke questionnaire (PILS-Stroke). This tool, which is still under development, will generate linear measures (in percentage of the full range scale expressed in logits), from 0% (low participation) to 100% (high participation).

##### Personal Factors

Confidence after a stroke will be evaluated using the 27-item Confidence After Stroke Measure. This self-administered questionnaire consists of 9 items of self-confidence (ie, feeling confident in one’s abilities, qualities, and judgment), 8 items of positive attitudes (ie, being optimistic about situations, interactions, and oneself), and 10 items of social confidence (ie, feeling comfortable in social situations and in social acceptance). Each item is rated on a 4-level scale, leading to a total score ranging from 27 to 108 points. The higher the values, the higher the confidence. The Confidence After Stroke Measure has shown good psychometric properties [49].

##### Health-Related Quality of Life

Health-related quality of life after stroke will be evaluated using the 59-item Stroke Impact Scale (SIS) [50]. This self-reported questionnaire covers 8 main health-related quality of life domains: strength (4 items), hand function (5 items), (instrumental) activities of daily living (10 items), mobility (9 items), communication (7 items), emotion (9 items), memory/thinking (7 items), and participation/role function (8 items). Patients will be asked to estimate the difficulty they experience in completing each item on a 5-level scale. The SIS shows adequate to excellent reliability [51] and excellent validity [52]. The proxy-patient version of the SIS will also be completed by the caregiver [53]. For each domain, an ordinal score is calculated with the following formula: Transformed Scale = [(Actual raw score - lowest possible raw score) ÷ Possible raw score range] x 100. A composite physical subscale can be calculated by combining 4 of the physical domains.

##### Feasibility and Adherence to Treatment (Satisfaction Questionnaire)

A satisfaction questionnaire was developed to assess the feasibility of both the 2-week HABIT-ILE@home intervention and the 9-week HABIT-ILE@home follow-up, and to compare them with the HABIT-ILE on-site camp and nonspecific follow-up, respectively. Patients’ and caregivers/interventionists’ adherence to treatment will also be recorded through the satisfaction questionnaire. The development of this questionnaire was informed by the HABIT-ILE@home pilot study, in which the feasibility, acceptability, and perceived burden of the telerehabilitation approach were assessed using instruments derived from validated scales [54]. Specifically, the questionnaire integrates dimensions adapted from the System Usability Scale, the User Experience Questionnaire, AttrakDiff2, the Dimensions of Mastery Questionnaire, the Intrinsic Motivation Inventory, the Exercise Adherence Rating Scale, and the Exercise Therapy Burden Questionnaire. Based on this, the questionnaire used in the present trial assesses the following dimensions: REAtouch Lite interactive system features, caregivers’ ability to follow the therapy schedule, and to apply motor skill learning principles, caregivers’ compliance, patients’ motivation and involvement, patients’ compliance (perceived usefulness of the therapy, enjoyment, and burden of the therapy), and preferences regarding therapy modalities (remote, in-person, or hybrid). The same questionnaire structure was adapted for the on-site modality, ensuring comparability (eg, removal of virtual-related sections, rewording of items). The satisfaction questionnaire will be filled in by patients, caregivers, and/or interventionists (only for on-site at T1), at the end of each intervention phase (T1 and T2).

##### Documentation of the Therapy Content

The content of the 2-week intensive therapy will be documented using multiple methods (RCT1). First, inertial measurement units, specifically the Xsens Dot (Xsens Technologies BV), will be used to measure and document motor activity (quantity and type of movements) during both on-site camp and at-home HABIT-ILE modalities. Each participant will be equipped with three wearable sensors during the 6.5 hours of daily high-dosage therapy: two placed dorsally on the wrists and one on the front of the right thigh. Activity count (based on a predefined threshold for movement) and asymmetry ratio (comparing right and left upper extremities activity) will be calculated and should make it possible to differentiate between uni- and bimanual tasks [55]. Accelerometer data should also make it possible to evaluate participants’ positions to determine whether they are predominantly sitting or standing during therapy sessions, as well as to quantify the percentage of time spent walking. However, inertial measurement unit–based classification remains approximate, and specific tasks cannot be distinguished with certainty. During the on-site modality, interventionists will complete a structured timesheet detailing each activity in terms of duration and type of motor activity performed throughout the day. For the home modality, therapy content will be partly documented based on the prescribed daily program and data extracted from the REAtouch Lite system (session duration and touch count on the screen). Additionally, at the end of each day, participants will report the duration of therapy they were able to complete.

For the follow-up interventions (RCT2), participants will maintain a weekly log over a 9-week period, briefly describing the type and duration of activities performed each day. Monitoring the completion rates of therapy sessions and comparing the hours of therapy actually performed with those planned will enable us to judge the feasibility of setting up such follow-ups.

### Statistical Analysis

#### Overview

Statistical analysis will be performed after the last measurements at the follow-up assessment using RStudio software (version 2023.12.15+402; Posit). Baseline characteristics will be summarized with means and SDs for normally distributed variables, medians and IQRs for nonnormal variables. Baseline group equivalence will be explored descriptively using independent *t* tests or nonparametric equivalents.

#### RCT 1: HABIT-ILE@home vs HABIT-ILE on-Site Camp (2 Weeks)

Non-inferiority statistics will be used to compare the efficacy of the HABIT-ILE@home modality with the HABIT-ILE on-site camp modality. First, within each intervention arm, changes from baseline (T0) to postintervention (T1) will be evaluated for all outcomes. Depending on data normality, paired samples *t* tests or nonparametric equivalents will be applied. Then, the 95% CI of the difference between groups will be computed by comparing group differences in change scores using independent *t* tests or nonparametric equivalents. Noninferiority will be concluded if the lower bound of this interval is above the prespecified NIM. For all outcomes, NIM will be defined as 50% of the difference between the changes observed with a gold standard (ie, CIMT) and a control group [13].

#### RCT 2: HABIT-ILE@home vs Nonspecific Follow-Up Interventions (9 Weeks)

Superiority statistical analyses will be used to examine changes during the 9-week follow-up period (T2-T1). Depending on the distribution of the data, the analyses will be conducted using either analysis of covariance (ANCOVA; for normally distributed data) or nonparametric equivalents (eg, rank-based ANCOVA) for nonnormally distributed data. Covariates such as initial T1 scores, mRS, and/or type of initial intervention will be included in the model to adjust comparisons. In case of analyses including all three time points, linear mixed-effects models will be used, with baseline score and group allocation entered as fixed effects.

#### Feasibility and Adherence

For questionnaire items shared by both groups, between-group comparisons will use Wilcoxon rank-sum tests. Items specific to the home-based condition will be summarized descriptively.

#### Missing Data

All analyses will follow an intention-to-treat approach. Missing data will be addressed using multiple imputation. All variables and auxiliary variables predictive of missingness will be included in the imputation model.

#### Clinical Significance

The proportion of participants exceeding established minimal clinically important differences on each outcome will be reported for both groups.

## Results

Recruitment started in March 2023 and ended in March 2025. All participants have been recruited and have undergone interventions in RCT1 and RCT2. Data analysis is expected to start in spring 2026.

## Discussion

### Principal Findings

Previous studies have shown that home-based telerehabilitation may be as effective as in-person therapies [56] with good treatment adherence [57]. Specifically, intensive interventions based on motor skill learning principles such as CIMT have shown similar effectiveness at home (both with or without a rehabilitation device) compared to in-person CIMT [19] or standard therapy [58]. Based on these previous results, we anticipate similar improvements when comparing HABIT-ILE@home and HABIT-ILE on-site camp within the first RCT.

However, there may be some challenges for participants to engage in such an intensive home intervention (short duration, high dosage), or even for a home follow-up intervention (longer duration, low to moderate dosage). The higher dosage of the therapy (65 hours) compared to previous studies may be more difficult for participants and caregivers to manage in terms of tiredness and motivation. Nevertheless, intrinsic motivation developed by participants based on the self-determined functional goals and the perceived usefulness of the therapy might help to maintain their treatment compliance. Indeed, a HABIT-ILE@home pilot study conducted in 3 adults with chronic stroke showed that the perspective of improving their motor control capacities, reaching their goals, and gaining autonomy was their main source of motivation, more than the playful nature of the therapy [54]. The daily program sent each day by the supervision team will aim to target the functional goals considered as important by the participant, thus supporting their intrinsic motivation.

In addition to the patients’ and caregivers’ motivation and adherence to treatment, a second aspect of the home-based protocol that needs to be documented is the burden of treatment. Indeed, caring for patients with chronic stroke may impose a significant burden on caregivers and could lead to heightened levels of anxiety and depression [59]. This affected mental health in caregivers can result in slower progress and poorer overall outcomes for the patient [60]. Moreover, the emotional state of caregivers and stroke patients is interconnected. Patients whose caregivers report greater burdens often exhibit higher levels of anxiety themselves, which could also impact patient improvements [59,61].

Introducing a new rehabilitation approach that involves caregivers may initially increase their burden, as they must understand and adapt to new responsibilities associated with the therapeutic process [61,62]. However, over time, as caregivers gain competence in managing their relative’s health condition, they may experience reduced anxiety and greater satisfaction in their caregiving role [63,64]. This shift highlights the potential of involving the caregivers in rehabilitation to not only enhance the stroke patients’ recovery but also improve the mental health of the caregivers. Involving caregivers in rehabilitation can lead to mutual benefits for themselves and stroke patients, ultimately promoting better health outcomes. However, this can only be achieved if caregivers feel sufficiently supported by therapists by receiving adequate clinical supervision [65]. As planned in the present intervention, previous studies have observed a positive effect of a dedicated initial training to reduce the caregivers’ burden and enhance perceived competence in managing therapeutic tasks, resulting in less stress and a greater engagement in rehabilitation [66].

Based on the current recommendations for motor and functional stroke rehabilitation, we hypothesize that the specific HABIT-ILE@home follow-up will result in better retention of changes following 2-week high-dosage therapies, and even better additional improvements than the nonspecific follow-up intervention (RCT 2) [67,68]. The HABIT-ILE follow-up program follows these recommendations by using a patient-centered approach that focuses on setting individualized goals to shape the therapy. It encourages self-initiated movements through goal-oriented training, promoting motor learning through trial and error. The program also incorporates shaping techniques, where activities are adapted to the patient’s current abilities and gradually become more challenging as they progress. This is further supported by intensive practice sessions designed to maximize the number of repetitions. However, adding 5 hours of home therapy per week to the participants’ usual schedule may be a challenge. Multiple determinants may cause nonadherence to a home-based program: psychological factors, lack of social support, time constraints, and complexity of activities proposed [69,70]. To avoid nonadherence to the prescribed exercise regimen during HABIT-ILE@home follow-up, we plan to set up a weekly schedule of exercises tailored to individual goals of each participant along with gamified activities in addition to the 1-hour supervised session each week [71].

The participants’ adherence to the 2-week HABIT-ILE@home intervention and the 9-week HABIT-ILE@home follow-up, as well as the burden and feasibility of these interventions, will be documented through a satisfaction questionnaire. This should help us highlight some of the pros and cons of such interventions. Another concern that will be investigated is the possibility of adhering to the HABIT-ILE therapeutic principles during the whole therapeutic time in the home-based setting. This will be partially inferred from the accelerometers, REAtouch Lite, and logbook data documenting the content of the therapy. Even if we cannot be sure of the correct application of all the HABIT-ILE principles, we assume that caregivers’ and participants’ training before therapy, provision of a daily detailed treatment schedule, and adequate remote supervision will help to address this concern.

This trial is expected to determine whether HABIT-ILE@home can produce improvements comparable to those obtained during the on-site intensive program across motor, activity, and participation-related outcomes, while substantially reducing logistical and accessibility barriers. Demonstrating noninferiority would support the scalability of high-dose bimanual motor-learning interventions in community settings. The follow-up phase will also provide important insights into the role of structured, task-specific home programs in maintaining or enhancing gains achieved during intensive interventions. If the specific HABIT-ILE@home follow-up proves superior to the nonspecific program, this would highlight the importance of targeted postintensive rehabilitation to promote retention and transfer of improvements into daily life.

### Conclusion

This study will provide evidence on the feasibility and efficacy of delivering HABIT-ILE through a home-based telerehabilitation modality for adults with chronic stroke. Demonstrating noninferiority of HABIT-ILE@home compared to on-site therapy would support wider accessibility to intensive rehabilitation while reducing logistical and human resource constraints. Additionally, showing the added benefit of a structured follow-up could emphasize the importance of continuity of care to sustain and enhance motor recovery after intensive interventions.

## Supplementary material

10.2196/87035 Checklist 1 SPIRIT checklist.

## Abbreviations

ACTIVLIM: Activity Limitations Questionnaire
ANCOVA: analysis of covariance
CIMT: constraint-induced movement therapy
FMA: Fugl-Meyer Assessment
FMA-UE: upper extremity subscale of the Fugl-Meyer Assessment
HABIT-ILE: hand and arm bimanual intensive therapy including lower extremities
HADS: Hospital Anxiety and Depression Scale
Mini-BESTest: Mini Balance Evaluation Systems Test
mRS: modified Rankin Scale
NIM: noninferiority margin
PILS-Stroke: Participation In Life Situations – Stroke questionnaire
RCT: randomized controlled trial
REDCap: Research Electronic Data Capture
SIS: Stroke Impact Scale
WMFT: Wolf Motor Function Test
